# Assessment of Exposure to Environmental Toxins and Racial and Ethnic Disparities in COVID-19 Hospitalizations Among US Veterans

**DOI:** 10.1001/jamanetworkopen.2022.24249

**Published:** 2022-07-28

**Authors:** Michelle S. Wong, W. Neil Steers, Stephen Frochen, Donna L. Washington

**Affiliations:** 1 Veterans Affairs Health Services Research and Development Center for the Study of Healthcare Innovation, Implementation, and Policy, Veterans Affairs Greater Los Angeles Healthcare System, Los Angeles, California; 2 Division of General Internal Medicine and Health Services Research, Department of Medicine, Geffen School of Medicine, University of California, Los Angeles, Los Angeles

## Abstract

This cohort study examines whether exposure to environmental toxins mediates the association of racial and ethnic disparities with hospitalizations among US veterans with COVID-19.

## Introduction

Residential neighborhood social factors associated with health play a role in the disproportionate impact of the COVID-19 pandemic among racial and ethnic minority populations.^1^ Exposure to environmental toxins has been understudied as a relevant neighborhood factor associated with health. Structural racism has relegated racial and ethnic minority populations to residential communities with higher, and multiple, risks of exposure to environmental toxins (eg, communities near industrial facilities).^2^ Although this exposure may be associated with increases in COVID-19–related comorbidities (eg, cancers),^3,4^ which might explain some of the racial and ethnic disparities in outcomes, it is unknown whether exposure to environmental toxins is independently associated with increases in COVID-19 susceptibility. This cohort study examined the hypothesis that exposure to environmental toxins mediates the association of racial and ethnic disparities with hospitalizations among veterans with COVID-19 after accounting for individual-level risk factors and comorbidities.

## Methods

We linked data on veterans with COVID-19 between March 1, 2020, and September 30, 2021, from the Veterans Health Administration national COVID-19 Shared Data Resource to data on exposure to environmental toxins from the US Environmental Protection Agency 2019 risk-screening environmental indicators (RSEI) model,^5^ which provided estimates of exposure for each census tract in the aggregate census tract data file. The study was approved by the institutional review board of the Veterans Affairs Greater Los Angeles Healthcare System and was deemed exempt from the need for informed consent because the analysis involved no more than minimal risk to participants and could not be carried out practically without the waiver. This study followed the Strengthening the Reporting of Observational Studies in Epidemiology (STROBE) reporting guideline for cohort studies,

Our dependent variable was hospitalization, and our main independent variable was race and ethnicity. For our mediating variable, exposure to environmental toxins, we used the RSEI score,^5^ which accounts for the amount of toxic chemicals released, the potential route of human exposure, each chemical’s relative toxicity, and the number of people potentially exposed. Higher RSEI scores indicate higher risk of toxic exposure; in this study, scores were classified into deciles. Control variables included age, sex, individual socioeconomic status, COVID-19 pandemic period, presence of chronic lung disease, presence of cancer, and smoking status (eMethods in the Supplement).

### Statistical Analysis

We used the Tchetgen Tchetgen inverse odds ratio (OR)–weighted regression method to assess mediation (eMethods in the Supplement).^6^ We then estimated indirect (mediated) effects by subtracting direct effect coefficients from total effect coefficients for each non-Hispanic White racial and ethnic group and bootstrapping the estimates. Data were analyzed using Stata software, version 17.0 (StataCorp LLC). We considered 2-tailed *P* < .05 to be statistically significant.

## Results

Among 286 781 veterans with COVID-19, 44.8% were younger than 60 years, and 89.4% were male. Overall, 0.8% of veterans were American Indian or Alaska Native, 1.0% were Asian, 10.4% were Hispanic, 0.8% were Native Hawaiian or other Pacific Islander, 22.4% were non-Hispanic Black, and 64.7% were non-Hispanic White (Table 1). Veterans who were hospitalized (15.3%) lived in neighborhoods with higher RSEI scores (mean [SD], 53.9 [2.9]) than those who were not hospitalized (mean [SD], 50.9 [2.9]). Non-Hispanic Black veterans had the highest proportion of hospitalizations (19.0%) and lived in neighborhoods with the highest RSEI scores (mean [SD], 59.5 [26.8]).

**Table 1.** Characteristics of Veterans With COVID-19 in the US Veterans Health Administration

Characteristic	Veterans, No. (%)		
	Total	Hospitalized	Not hospitalized
Total veterans, No.	286 781	43 893	242 888
Race and ethnicity			
American Indian or Alaska Native	2290 (0.8)	364 (0.8)	1926 (0.8)
Asian	2766 (1.0)	310 (0.7)	2456 (1.0)
Hispanic	29 683 (10.4)	3894 (8.9)	25 789 (10.6)
Native Hawaiian or other Pacific Islander	2263 (0.8)	308 (0.7)	1955 (0.8)
Non-Hispanic Black	64 310 (22.4)	12 225 (27.9)	52 085 (21.4)
Non-Hispanic White	185 469 (64.7)	26 792 (61.0)	158 677 (65.3)
Age range, y			
<60	128 354 (44.8)	10 596 (24.1)	117 758 (48.5)
60-64	28 889 (10.1)	4884 (11.1)	24 005 (9.9)
65-69	28 774 (10.0)	5670 (12.9)	23 104 (9.5)
70-74	49 480 (17.3)	9909 (22.6)	39 571 (16.3)
75-79	24 290 (8.5)	5484 (12.5)	18 806 (7.7)
≥80	26 993 (9.4)	7349 (16.7)	19 644 (8.1)
Sex			
Female	30 356 (10.6)	2495 (5.7)	27 861 (11.5)
Male	256 425 (89.4)	41 398 (94.3)	215 027 (88.5)
Individual socioeconomic status^a^			
High	38 408 (13.4)	5303 (12.1)	33 105 (13.6)
Low	46 624 (16.3)	10 668 (24.3)	35 956 (14.8)
Indeterminate	201 522 (70.3)	27 898 (63.6)	173 624 (71.5)
COVID-19 pandemic period			
Early pandemic^b^	14 228 (5.0)	3453 (7.9)	10 775 (4.4)
Summer surge^c^	38 109 (13.3)	6178 (14.1)	31 931 (13.1)
Winter surge^d^	145 565 (50.8)	19 863 (45.3)	125 702 (51.8)
Vaccines available^e^	29 958 (10.4)	4555 (10.4)	25 403 (10.5)
Delta variant surge^f^	58 920 (20.5)	9843 (22.4)	49 077 (20.2)
Chronic lung disease	82 617 (28.8)	17 724 (40.4)	64 893 (26.7)
Cancer	49 582 (17.3)	11 686 (26.6)	37 896 (15.6)
Smoking status			
Current	36 808 (12.8)	5847 (13.3)	30 961 (12.7)
Former	117 450 (41.0)	19 752 (45.0)	97 698 (40.2)
Never	108 877 (38.0)	14 444 (32.9)	94 433 (38.9)
Unknown	23 646 (8.2)	3850 (8.8)	19 796 (8.2)
RSEI percentile, mean (SD)	51.4 (2.9)	53.9 (2.9)	50.9 (2.9)

Abbreviation: RSEI, risk-screening environmental indicators.

^a^ Socioeconomic status was defined based on Veterans Health Administration enrollment priority group. High socioeconomic status was defined as having an income higher than the Veterans Health Administration threshold for requiring copayment and low socioeconomic status as having an income lower than this threshold. Individual socioeconomic status was not available for veterans with service-connected disability; these veterans were classified in the indeterminate category.

^b^ March 1 to May 31, 2020.

^c^ June 1 to September 30, 2020.

^d^ October 1, 2020, to February 28, 2021.

^e^ March 1 to June 30, 2021.

^f^ July 1 to September 30, 2021.

Higher RSEI scores were associated with increased hospitalization (OR, 1.04; 95% CI, 1.03-1.04). After covariate adjustment, all racial and ethnic minority groups had higher odds of hospitalization compared with non-Hispanic White veterans (eg, total effect for non-Hispanic Black veterans: OR, 1.66; 95% CI, 1.62-1.71) (Table 2). After accounting for comorbidities, RSEI decile scores mediated racial and ethnic hospitalization disparities among Hispanic and non-Hispanic Black veterans, accounting for 4.43% of the disparity among Hispanic veterans and 3.26% of the disparity among non-Hispanic Black veterans vs non-Hispanic White veterans.

**Table 2.** Inverse Odds Ratio–Weighted Mediation of Toxic Environmental Exposure in the Association of Racial and Ethnic Disparities With Hospitalizations Among US Veterans With COVID-19^a^

Race and ethnicity	OR (95% CI)			Percentage attributable to mediator
	Total effect	Direct effect	Indirect effect	
American Indian or Alaska Native	1.35 (1.18-1.51)	1.31 (1.15-1.48)	1.03 (0.99-1.06)	10.49
Asian	1.27 (1.11-1.43)	1.28 (1.10-1.42)	1.01 (0.99-1.03)	4.43
Hispanic	1.31 (1.25-1.36)	1.29 (1.24-1.35)	1.01 (1.00-1.02)	4.43
Native Hawaiian or other Pacific Islander	1.18 (1.03-1.34)	1.19 (1.04-1.34)	1.00 (0.92-1.01)	−2.99
Non-Hispanic Black	1.66 (1.62-1.71)	1.64 (1.60-1.69)	1.01 (1.01-1.02)	3.26
Non-Hispanic White	1 [Reference]	1 [Reference]	1 [Reference]	NA

Abbreviations: NA, not applicable; OR, odds ratio; RSEI, risk-screening environmental indicators.

^a^ Toxic environmental exposure was measured by RSEI scores and converted to deciles, with ORs and the percentage possibly explained by the mediator corresponding to the percentage explained per decile increase in RSEI score. Models controlled for age (<60 years, 60-64 years, 65-69 years, 70-74 years, 75-79 years, or ≥80 years), sex (female or male), individual socioeconomic status (low, high, or indeterminate), COVID-19 pandemic period (early pandemic [March 1 to May 31, 2020], summer surge [June 1 to September 30, 2020], winter surge [October 1, 2020, to February 28, 2021], vaccines available [March 1 to June 30, 2021], Delta variant surge [July 1 to September 30, 2021]), presence of chronic lung disease, presence of cancer, and smoking status (current, former, or never). Analyses that included chronic lung disease, cancer, and smoking status as mediators along with exposure to environmental toxins (eMethods in the Supplement) revealed overall indirect effects that were similar in extent to the indirect effects found when including exposure to environmental toxins as the only mediator.

## Discussion

In this cohort study, exposure to environmental toxins partially mediated the association of racial and ethnic disparities with COVID-19 hospitalizations, independently of COVID-19–related comorbidities. Exposure to environmental toxins may weaken the respiratory and immune systems of exposed individuals, who disproportionately belong to racial and ethnic minority populations, increasing their susceptibility to serious COVID-19 independently of comorbidities and tobacco use. Limitations of this study include its limited generalizability to children, lack of accounting for other neighborhood factors associated with health (eg, poverty), potential for measurement error (eg, participants’ residential histories and mobility), and possibility of being underpowered to detect mediation in smaller racial and ethnic minority groups.

Future research may examine exposure to specific environmental toxins. Addressing racial and ethnic health disparities requires equity-focused planning and environmental policies that address pollution and promote environmental justice for racial and ethnic minority communities. Advocating for environmental justice may also aid future disaster-preparedness efforts.

## Notes

Supplement. **eMethods.** Study Population, Measures, and Statistical Analysis
eReferences
