# Supplementary material for: Assessment of Exposure to Environmental Toxins and Racial and Ethnic Disparities in COVID-19 Hospitalizations Among US Veterans
Source: JAMA Netw Open. 2022 Jul 28;5(7):e2224249. doi: 10.1001/jamanetworkopen.2022.24249 (PMC9335138; doi:10.1001/jamanetworkopen.2022.24249)
Supplement: Supplement. — eMethods. Study Population, Measures, and Statistical Analysis eReferences [file jamanetwopen-e2224249-s001.pdf]

Supplementary Online Content

Wong MS, Steers WN, Frochen S, Washington DL. Assessment of exposure to environmental toxins and racial and ethnic disparities in COVID-19 hospitalizations among US veterans. *JAMA Netw Open*. 2022;5(7):e2224249. doi:10.1001/jamanetworkopen.2022.24249

eMethods. Study Population, Measures, and Statistical Analysis

eReferences

This supplementary material has been provided by the authors to give readers additional information about their work.

© 2022 Wong MS et al. *JAMA Network Open*.

eMethods. Study Population, Measures, and Statistical Analysis

Study population.

Our sample was comprised of veterans who tested positive for COVID-19 based on COVID-19 polymerase chain reaction tests of nasopharyngeal swab specimens and COVID-19 antigen tests conducted in VA as well as the community. All 50 states, the District of Columbia, and U.S. territories were all represented in our sample, with the largest proportion residing in the Southern Region (46%), followed by Midwest (22%), West (18%), and Northeast (11%).

Measures.

For the outcome variable, hospitalization, we included hospitalizations at VA facilities related to COVID-19.

For control variables, we obtained individual socio-economic status (SES) based on Veteran Health Administration's (VHA's) enrollment priority group. Since VHA does not collect income information for veterans with service-connected disability, we could not determine individual SES for these individuals. COVID time periods corresponded to peaks and troughs in the pandemic. We obtained chronic lung disease, cancer, and smoking status from electronic medical record data two years prior to patients' positive test date. We controlled for chronic lung disease and cancer because these comorbidities may be caused by exposure to environmental toxins and are risk factors for COVID-19 hospitalizations.[1,2] Being a smoker may exacerbate the effect of exposure to environmental toxins on COVID-19.

Statistical analysis.

We fit a fully-adjusted logistic regression model to determine whether RSEI scores were associated with the hospitalization outcome.

We tested mediation using the Tchetgen Tchetgen method involving inverse odds-ratio (IOR)-weighted regression models as described by Nguyen 2015's practical guide[3]. The method involves decomposing the total effect of race and ethnicity on hospitalization into its direct (unmediated) and indirect (mediated) effect components. We first estimated the total effect of race and ethnicity on hospitalization using logistic regression while adjusting for all covariates listed above and exposure to environmental toxins. Next, we estimated the direct effect of race/ethnicity on hospitalization through a two-step process. We first obtained the IOR weights by fitting a polytomous regression model predicting race and ethnicity from RSEI decile scores with covariate adjustment, and subsequently transformed the race and ethnicity coefficients into IOR weights. Non-Hispanic White veterans were given a weight of 1 since they were the reference group, while veterans from each racial and ethnic minority group were given the corresponding IOR weight. In the second step, we fit an inverse odds-ratio-weighted logistic regression model to estimate the direct effect of race and ethnicity on hospitalization while controlling for the same covariates as from the total effects model. Finally, we obtained

© 2022 Wong MS et al. *JAMA Network Open*.

the indirect effects by subtracting the direct effect coefficients from the total effect coefficient for each racial and ethnic group. To aid interpretation, we transformed the total, direct, and indirect effect coefficients into odds ratios (OR) and calculated the proportion of the total effect explained by the RSEI score mediator (OR^{NDE} (OR^{NIE}-1) / (OR^{NDE}*OR^{NIE} – 1)). Each of these estimates was bootstrapped to obtain standard errors and 95% confident intervals (CI).

We conducted two sensitivity analyses. In the first sensitivity analysis, we also added chronic lung disease, cancer, and smoking status as mediators, along with exposure to environmental toxins. One of the strengths of the Tchetgen Tchetgen inverse odds ratio weighting mediation approach is that it accommodates multiple mediators.[3] We followed the approach described above. The results showed an overall indirect effect whose magnitude was similar to the indirect effect mediated by exposure to environmental toxins that we found when we included exposure to environmental toxins as the only mediator in the model.

In the second sensitivity analysis, we included an interaction between the comorbidities and exposure to environmental toxins when creating the IOR weights. This occurring during the step when we ran a polytomous regression model predicting race and ethnicity from the mediators. We then proceeded with the remaining steps as described above. Including these interactions when creating the weights did not change our results or inferences.

© 2022 Wong MS et al. *JAMA Network Open*.

eReferences

1. Kampa M, Castanas E. Human health effects of air pollution. *Environmental Pollution.* 2008;151(2):362-367.
2. Centers for Disease Control and Prevention. Underlying Medical Conditions Associated with Higher Risk for Severe COVID-19: Information for Healthcare Professionals. 2022; https://www.cdc.gov/coronavirus/2019-ncov/hcp/clinical-care/underlyingconditions.html. Accessed February 11, 2022.
3. Nguyen QC, Osypuk TL, Schmidt NM, Glymour MM, Tchetgen Tchetgen EJ. Practical Guidance for Conducting Mediation Analysis With Multiple Mediators Using Inverse Odds Ratio Weighting. *American Journal of Epidemiology.* 2015;181(5):349-356.

© 2022 Wong MS et al. *JAMA Network Open*.
